# Type II polyketide synthases: Impact on human health, current knowledge, and future directions

**DOI:** 10.1016/j.jbc.2025.110749

**Published:** 2025-09-22

**Authors:** Jacob T. Wolff, Shiou-Chuan Tsai

**Affiliations:** 1 Department of Molecular Biology and Biochemistry, University of California, Irvine, California, USA; 2 Department of Pharmaceutical Sciences, University of California, Irvine, California, USA; 3 Department of Chemistry, University of California, Irvine, California, USA

**Keywords:** polyketide, acyltransferase, acyl carrier protein (ACP), protein-protein interaction, natural product biosynthesis, ketoreductase, ketosynthase, aromatase/cyclase

## Abstract

Natural products have a long history of use in traditional and modern medicine due to their inherent bioactivity. Some medicinal activities include antibiotic, antifungal, anticancer, antiviral, antihypercholesterolemic, and immunosuppressant. One of the largest classes of bioactive natural products are polyketides, produced by polyketide synthases (PKSs). PKSs are closely related to fatty acid synthases, sharing a core biosynthetic logic that iteratively builds larger molecules from simple precursors. However, PKS produce compounds with incredible structural diversity and function through the accretion of small chemical alterations not available to fatty acid synthase at each point during synthesis. Polyketide biosynthesis can be grouped into initiation, extension, reduction, aromatization and cyclization, and tailoring steps. Changes at each step have the potential to produce many variations in structure motivating prodigious research efforts to understand and engineer new PKS that produce novel medicinal compounds. Despite success creating chimeric PKS that produced new compounds, yield and fidelity were decreased, and these successes have made clear that understanding protein-protein interactions is critical for improved engineering outcomes. In this review, we lay out of the importance of natural products assembled by type II PKSs in human health, and how these molecules are assembled, and conclude by summarizing the challenges currently facing the field.

## Why natural products?

Natural products (NPs) have long been a staple of human society and range from spices to life-saving drugs. Records of NPs used for medicine, mainly plants, reach as far back as 2600 B.C. in Mesopotamia and continue through the 18th and 19th centuries when plant extracts continued to yield new treatments (1, 2). Such a lengthy history highlights the long-standing importance of NPs to human health, but it was not until Alexander Fleming’s discovery of penicillin in 1928 coupled Penicillin’s effectiveness in World War II that NP drug discovery began in earnest (3). Large industrial companies including Merck, Squibb, Lilly, and Pfizer contributed to scaling up Penicillin production during the war and helped usher in the “Golden Age” of drug discovery that lasted from the 1940s to the 1970s (4, 5, 6).

This first era in the “Golden Age” was marked by collecting soil samples, attempting to culture the microbes present, extracting small molecules from the cells, and testing for activity using simple cell death assays (5, 6). If a compound induced cell death, purification, identification, and structural characterization followed. This approach continued into the 1970s through the 1990s augmented by new drug target assays, diversified NP sources, miniaturized assays, and improved dereplication using HPLC, mass spectrometry, and NMR to create fingerprints (5, 6, 7). In 1985, advancements in recombinant DNA led to the first cloning of an entire biosynthetic gene cluster (BGC) from the soil bacteria *Streptomyces coelicolor*, soon followed by mixing elements from other BGCs to create novel compounds (8, 9). This kicked off a frenzy of BGC cloning and further creation of Frankenstein BGCs, called “combinatorial biosynthesis” that resulted in producing novel compounds, but this was quickly overshadowed by the rise of combinatorial chemistry (1, 5, 6, 7, 10, 11, 12, 13, 14, 15, 16, 17, 18, 19, 20, 21, 22, 23, 24, 25).

Unfortunately, the immense high-throughput capacity of combinatorial chemistry failed to deliver on its promises with only two *de novo* compounds commercialized between 1981 and 2019 despite the millions of compounds screened (5, 26). A large part of this failure rested on poor library design, leading to a redesign of screening libraries for structural diversity, going as far as designing libraries to explore NP chemical space (1, 27, 28). Alongside this renewed focus on NP chemical space, NP discovery reemerged, driven by the wealth of metagenomic data available through low-cost DNA sequencing (29, 30, 31). This drove development of bioinformatics tools for BGC prediction and sequence databases that continues to this day (32, 33, 34, 35, 36, 37, 38). This predictive power cascaded into renewed development of techniques for DNA manipulation, host engineering and culture, compound extraction, and high-throughput NP screening and identification (27, 39, 40, 41, 42, 43).

**Figure 1:**
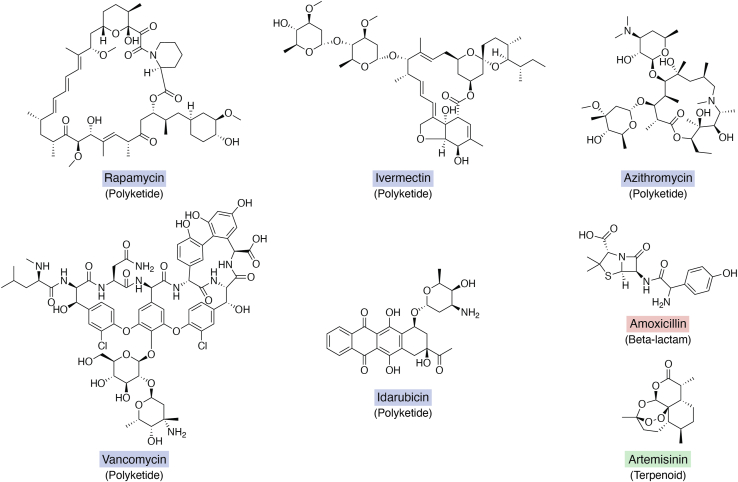
**Examples of bioactive polyketides, such as rapamycin (immunosuppressant), ivermectin (parasite treatment), azithromycin (antibiotics), vancomycin (antibiotics), artemisinin (antimalarial), idarubicin (leukemia treatment), and amoxicillin (antibiotics).** Polyketides have a huge molecular diversity in their chemical structures: starter units, chain lengths, ring formations, and ketoreduction patterns. The combination of the above factors leads to the huge polyketide diversity observed in nature.

The staying power that NPs have demonstrated over decades is most strongly evidenced by the fact that almost 70% of newly approved small molecule drugs from 1981 to 2019 come from or are inspired by NPs (26). Such drugs include azithromycin (from erythromycin), amoxicillin (from penicillin), cephalexin (from cephalosporin), vancomycin, daptomycin, ivermectin (from avermectin), artemisinin, idarubicin (from daunorubicin), and rapamycin (see (5) for more) (Fig. 1). These encompass antibacterial, antifungal, anticancer, antimalarial, and immunosuppressant activities, all important for modern medicine. Ivermectin, the derivative of polyketide avermectin, stands out as a testament to NP impact on human society, covering human, animal, and agricultural health.

Originally isolated from a soil bacteria in 1973, ivermectin was quickly identified to have anthelmintic function in 1974, and the more effective derivative ivermectin was commercialized in 1981 (44). A few years later, in 1987, ivermectin was found effective for treating river blindness, and later lymphatic filariasis, and was offered free of charge through Merck’s Mectizan donation program (45). Since its inception, the program has donated five billion treatments as of 2025, helping eliminate river blindness in five countries and lymphatic filariasis in another five (46). The next most significant impact of ivermectin has been for animal health as it is effective against multiple parasites, leading to sales of over one billion USD annually for over 20 years (47).

Considering the effectiveness of ivermectin across multiple diseases and across decades, along with room to expand in treating more diseases, it is the shining star of NP impact on medicine, especially polyketides (44). Given this success, understanding how these molecules are biosynthesized so that promising novel bioactive molecules can be produced through engineered biosynthesis is paramount to continued advancement. For the remainder of this review, we will turn our attention to the enzymes responsible for polyketide biosynthesis, since their biosynthetic logic, combined with similarity to fatty acid biosynthesis, provides a strong platform for novel molecule production. Specifically, we will focus on type II PKSs, whose molecular logic is much less understood.

## PKS architecture and biosynthetic logic

Polyketides are an important class of diverse NPs that are synthesized by enzyme complexes called polyketide synthases (PKSs). Molecules produced by PKS cover a wide range of bioactivity, including the antibiotics tetracycline, tetracenomycin, and actinorhodin; anticancer compounds like resistomycin, doxorubicin, and mithramycin; anti-fungals like pradimicin and amphotericin; and anti-HIV therapeutics rubromycin and griseorhodin (1, 2, 3, 4, 5, 6, 9, 13, 14, 44, 45, 46, 47, 48, 49, 50, 51, 52, 53, 54, 55). Despite the wide variety of polyketide structures produced, PKS share biosynthetic logic and enzymatic architecture with fatty acid synthases (FASs), which produce less complicated, mostly linear reduced hydrocarbon chains (56, 57, 58). The shared biosynthetic logic between FAS and PKS consists of initiation, extension, and reduction. Additional biosynthetic logic unique to PKS consist of aromatization/cyclization and tailoring, which are possible due to different levels of reduction at the reduction step when compared to FAS and the presence of more enzymes that modify the polyketide backbone. How each of these biosynthetic phases play out during polyketide or fatty acid synthesis is determined by the enzymatic architecture of the PKS or FAS systems. Below, we will focus specifically on type II PKS *versus* type II FAS.

### PKS/FAS architectures and biosynthetic overview: a comparison between type I and type II systems

**Figure 2:**
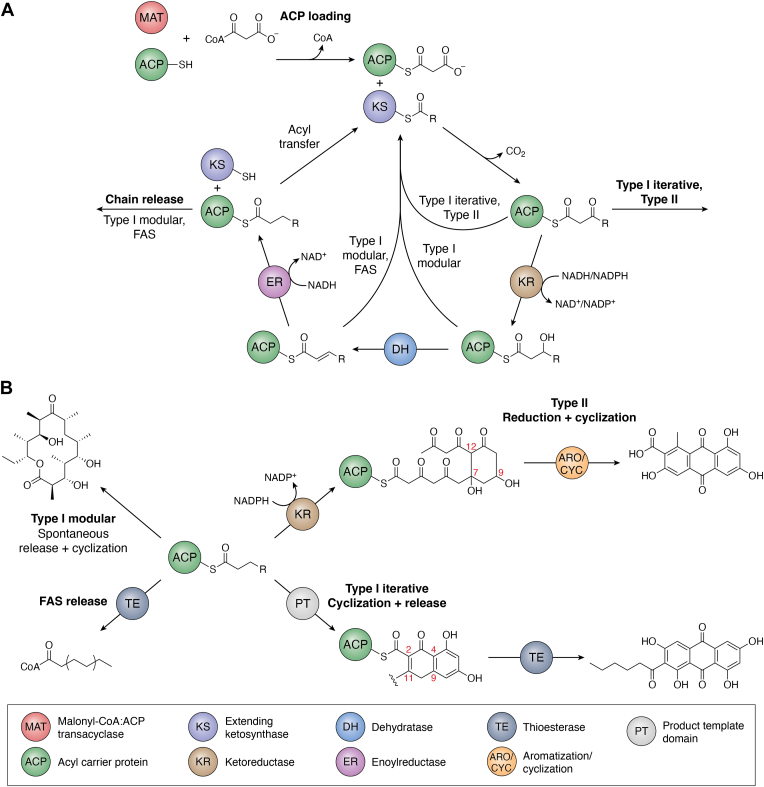
**A generalized biosynthetic scheme for type I and II PKSs.** Chain initiation is followed by chain elongation *via* the action of the ketosynthase (KS). Acyltransferase (AT, or malonyl acyltransferase, MAT) provides the extender units during chain elongation. During the biosynthetic process, the growing chain is attached to acyl carrier protein (ACP). This is followed by chain modifications such as ketoreduction and cyclization/aromatization (ARO/CYC). In fatty acid synthase, the growing chain is fully reduced to a simple carbon chain after each round of elongation, ketoreduction, dehydration, and enoyl reduction. In contrast, in polyketide synthase, type I modular PKS modifies the chain differently during each elongation round, whereas type II aromatic PKS build the chain to certain length then undergo one highly specific ketoreduction (shown in the *square* using the actinorhodin PKS as an example, with C7-C12 first ring cyclization and C9 ketoreduction). In addition, the fungal type I iterative PKS used a similar logistics as the type II bacterial PKS (in the *square*), achieving a certain chain length followed by cyclization catalyzed by the product template (PT) domain and chain termination by the thioesterase (TE). PKS, polyketide synthase; ARO/CYC, aromatase/cyclase.

PKS and FAS share similar architectures in type I and type II FAS and PKS. There is also a third architecture, type III that is only observed in PKS systems (Fig. 2). Type I PKS and FAS are large polypeptide chains that consist of multiple domains linked covalently together that can further be divided into type I iterative (highly reducing or nonreducing) and type I modular systems. Type I iterative PKS has only one “module”; it uses each domain in the polypeptide chain one or more times as does type I FAS. In comparison, modular PKS typically contain multiple “modules”, groups of enzymatic domains, where each domain only acts once as the acyl carrier protein (ACP)-tethered intermediate passes through each module (56, 59, 60, 61, 62).

In contrast to type I architecture, type II architecture consists of monodomain or didomain polypeptides that are not covalently linked and interact transiently during biosynthesis (63). The final architecture, type III, is only observed in PKS systems and is a “simple” homodimer of ketosynthase (KS) capable of catalyzing extension, cyclization, and aromatization without the need for additional enzymatic partners or a carrier protein (64, 65). In this review, we will focus on the type II PKS systems.

Regardless of type I or II, chain initiation is the first step in polyketide and fatty acid synthesis, consisting of a decarboxylative Claisen condensation reaction between a priming unit and an extending unit (66, 67, 68, 69). Both the priming molecule and extending unit can vary, but the most common primer and extender are acetate and malonate, respectively (57, 68, 70, 71). The priming unit is transferred to a KS active site cysteine *via* a thioester linkage through a variety of means, which will be covered in a subsequent section. The malonate entry, on the other hand, is consistent across type I and II PKS and FAS systems. A malonyl-CoA:ACP transacylase (MAT or MCAT) or acyltransferase (AT) transfers malonyl from malonyl-CoA to a phosphopantetheine (PPT) prosthetic group attached to an (ACP (Fig. 2). ACP brings the malonyl moiety to the KS that catalyzes the condensation between the priming unit and malonyl-ACP.

In type II systems, chain initiation can be catalyzed by the extending ketosynthase/chain length factor (KS/CLF) heterodimer as in actinorhodin and tetracenomycin biosynthesis, or by a priming KS homodimer as observed in frenolicin and R1128 biosynthesis, where a nonacetate priming unit is used (10, 66, 72, 73). Similar to the frenolicin and R1128 systems, a priming KS is also used for FAS initiation in type II systems (74, 75, 76).

In comparison to the above type II systems, the type I systems do not have a specialized priming KS; rather, they rely on their “default” KS; therefore, nonacetate priming units in type I systems enter the biosynthesis in two different ways. In a modular system, a loading AT/ACP is responsible for transferring the priming unit to the KS active site in the first module (68). In contrast, the type I iterative systems have a specialized AT, a Starter unit:ACP transacylase (SAT) domain, which selects the priming unit and loads it onto ACP for transfer to the KS domain (69, 77).

After chain initiation, the biosynthetic logic diverges based on different architectures, though the reactions remain the same. Due to the nature of condensation, a ketone and a methyl group are always added to the growing chain. In FAS, this ketone is immediately reduced to an alcohol by a ketoreductase (KR), then to an alkene by a dehydratase, and finally a fully reduced alkane through an enoylreductase (57). Except in unsaturated fatty acid biosynthesis, wherein some reduction steps are skipped, the intermediate undergoes full reduction before transfer back to the KS active site for iterative extension and reduction to produce a full length fatty acid chain. In order of the most reduced to the least, highly reducing (HR) type I iterative PKS function most similarly to type I FAS, but with less reduction due to skipping some reduction steps, likely because of kinetic competition, and additional chemical modification (78). Type I modular PKS catalyzes less reduction than HR PKS, but it reduces based on the reductive domains present in each module (59, 79). The core logic for these three synthase types can be summarized as one step of extension followed varied subsequent reductive steps (with further modification in PKS systems) that are repeated until completion.

In contrast, nonreducing (NR) type I iterative and type II PKS catalyze repeated condensation until the carbon skeleton has reached full extension as controlled by the KS (NR type I iterative) or KS/CLF (type II) (Fig. 2) (80, 81, 82). This biosynthetic scheme produces a highly reactive poly-β-ketone intermediate that affords ready cyclization and lactonization to form well-defined structures, though additional enzymes are required to guide cyclization away from spontaneous patterns (16, 80, 81, 83, 84, 85). However, the more reduced polyketides are not without their potential for cyclization through the presence of spaced alkenes that enable Diels-Alder cycloadditions within the same chain (86, 87). It is precisely the controlled reduction by PKS, in contrast to the complete reduction by FAS that provides the basis for complex molecules with well-defined, bioactive structure. For the type II PKSs, such selective reduction/cyclization leads to a huge diversity of aromatic polyketide NPs.

Following reduction, cyclization, and aromatization takes place in type II PKSs. To critically compare type I and II PKSs, in all types of PKS the aromatization and cyclization of the linear polyketide chain define the carbon skeleton shape. Cyclization reactions in type I modular PKS are catalyzed by thioesterase (TE) lactonization, as exemplified by 6-deoxyerythronolide B or, unique to spinosyn, Diels-Alder cycloaddition catalyzed by a Diels-Alderase (59, 79, 87). Type I iterative PKS also share these cyclization reactions, but they are split between the HR and NR subtypes. HR type I iterative PKS cyclize through Diels-Alder cycloadditions as in lovastatin, equisetin, and solanapyrone, while NR type I employs a TE domain for final cyclization through a Dieckmann condensation rather than lactonization (80, 86, 88, 89, 90). The cyclization in NR type I iterative systems is similar to the cyclization performed in type II PKS, because both involve aldol condensations catalyzed by an enzyme with an internal cavity that guides the reactive intermediate into its catalytic position (Fig. 2) (91). In NR type I iterative systems the product template domain controls first and second ring cyclizations, while aromatase/cyclase enzymes (ARO/CYC) perform this function in type II PKS (20, 23, 80, 92, 93, 94, 95).

After the carbon skeleton is formed, the final stage is tailoring, where the carbon skeleton is further modified. These modifications can include glycosylation, methylation, epoxidation, oxidative rearrangement, halogenation, and further reduction (96). Due to the breadth of tailoring reactions which vary widely between individual PKS clusters, further discussion will not be included, and there are further reviews that can be read which provide extensive information (97, 98, 99, 100). Subsequent sections will provide more detailed information regarding the core biosynthetic steps in type II PKS.

### Biosynthetic logic: chain initiation in type II PKSs

**Figure 3:**
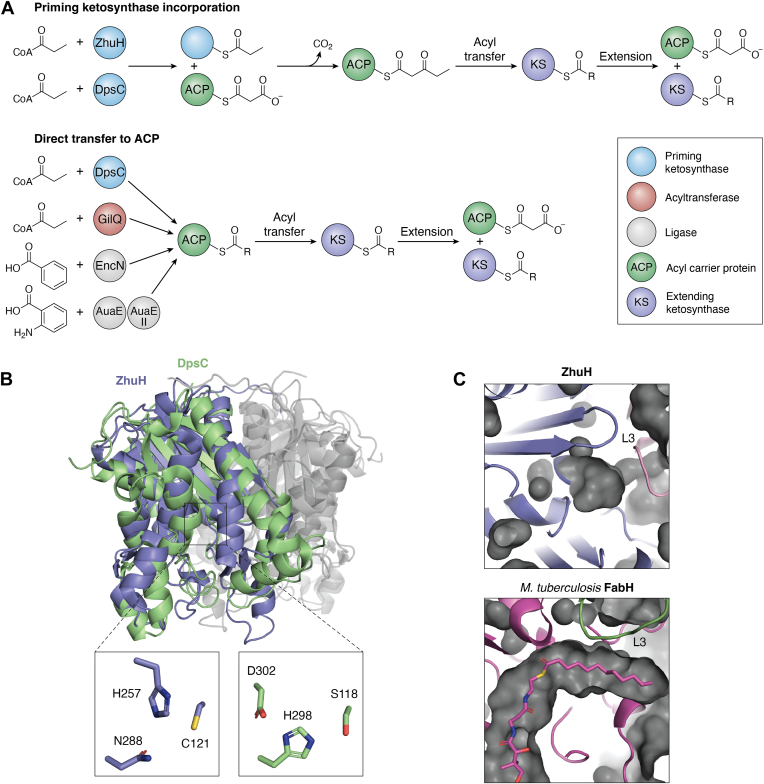
**The molecular logic of priming enzymes in type II PKS**. *A*, chemical reactions catalyzed by the priming ketosynthase (KS), and direct transfer of primer units to the ACP. *B*, a comparison of the priming KS, ZhuH (from the R1128 PKS), and DpsC (from the daunorubicin PKS), showing key differences in the active site triad. *C*, a comparison of the ZhuH substrate binding pocket with that of the fatty acid priming KS, FabH, from *Mycobacterium tuberculosis*. Note that in mtbFabH, T97 at the end of the pocket and near the dimer interface is important for chain length specificity. Pocket residues define the primer unit specificity, such that shorter ZhuH pocket accepts a small acyl unit, while FabH pocket accommodates the long fatty acyl priming unit. PKS, polyketide synthase; mtbFabH, Mycobacterium tuberculosis FabH; ACP, acyl carrier protein.

Chain initiation involved a priming unit that is condensed with an extending unit (Fig. 3*A*). In type II PKS, priming with acetate is commonly observed and is a result of malonyl decarboxylation catalyzed by the KS/CLF after malonyl transfer to the KS active site cysteine, as observed in actinorhodin and tetracenomycin biosynthesis (19, 66, 101, 102). However, there are also many type II systems that prime with a nonacetate priming unit, which adds significant diversity to the resulting polyketides. These nonacetate priming units include propionate (daunorubicin, aclacinomycin, lomaiviticin, and R1128A), butyrate (R1128B, frenolicin, and alnumycin), isobutyrate (R1128C), malonamide (oxytetracycline), hexanoate (benastatin), benzoate (enterocin), salicylate (thermorubin), and hexadienyl group (hedamycin and fredericamycin) (10, 103, 104, 105, 106, 107, 108, 109, 110, 111, 112). This assortment ranges from short, linear hydrocarbons (propionate and butyrate) to aromatic rings (benzoate and salicylate), thus providing a substantial source of diversity.

In type II PKSs, the systems that incorporate a nonacetate primer contain a so-called “initiation module” that contains one or more enzymes responsible for catalyzing incorporation. The most common enzyme is the priming KS, originally identified based on sequence similarity to FabH, the priming KS in *Escherichia coli* (11, 67, 113, 114). The role of the priming KS in promoting nonacetate priming units was first observed in the daunorubicin/doxorubicin-producing PKS through a combination of *in vivo* and *in vitro* experiments, where DpsC presence correlated with propionate priming (115, 116, 117, 118). This selective role was further confirmed during the study of ZhuH from the R1128 PKS, which showed preference for the propionate, but also primed acetyl-, butyryl-, and isobutyryl-CoA with the same proportion as the observed R1128 priming unit composition *in vivo* (73, 104, 113).

These two enzymes, ZhuH and DpsC, represent the two branches of priming KSs that differ in catalytic triad composition. The ZhuH catalytic triad consists of C121, H256, and N288, which matches the catalytic triad in FabH from *E. coli* and priming KSs from other type II FAS pathways (Fig. 3) (74, 119, 120, 121, 122, 123). FrnI, BenQ, and AlnI from the frenolicin-, benastatin-, and alnumycin-synthesizing systems are examples of additional FabH-like priming polyketide KSs (10, 106, 124). In contrast, DpsC contains the AT-like catalytic triad S118-H297-D302 and has been confirmed to effectively self-acylate and transfer acyl groups to its cognate ACP, a function not observed in FabH-like priming KS (Fig. 3*A*) (125). FdmS, HedS, and AknE2 from the fredericamycin-, hedamycin-, and aclacinomycin-synthesizing PKSs are additional DpsC-like priming KSs based on sequence similarity (111, 126, 127). Despite the distinct catalytic triad differences, structures of both ZhuH and DpsC are mostly in agreement, with a conserved thiolase fold that has an L-shaped active site entrance and acyl-binding pocket (Fig. 3*B*) (123, 125). Both structures are homodimers with a loop, L3 that extends from one monomer to the back of the active site in the other monomer, forming a “cap” on the acyl-binding pocket that appears to physically restrict the type of acyl groups that can be transferred to the active site nucleophile (125). This trend holds true for DpsC homologs based on sequence-substrate comparisons; however, the same relationship is not observed for FabH-like KSs.

Several FAS priming KS structures exist with ligands bound to the active sites, which elucidate how the acyl-binding pocket residues affect the selectivity for fatty acid priming unit. In *E. coli* FabH (ecFabH), the cap residue is F87, which was hypothesized to restrict ecFabH to favor acetyl-CoA based on bulk (76, 119, 128). Although reasonable, the *Acinetobacter baumannii* FabH (abaFabH), which has the structurally similar Y131, favors octanoyl-rather than acetyl-CoA (Fig. 3*C*) (129). This same contradiction is also borne out in the *Mycobacterium tuberculosis* FabH (mtbFabH), which is capped by a threonine residue (T97) as is ZhuH, yet favors lauroyl-CoA, a fourteen-carbon compound, rather than propionyl-CoA (73, 130, 131). Furthermore, the pockets in abaFabH and mtbFabH differ in configuration despite both favoring long chain acyl-CoAs (Fig. 3*C*). The octanoyl-bound abaFabH structure shows the acyl chain bending away from the Y131 cap, whereas T97 in mtbFabH enables the lauroyl to enter a hydrophobic cavity formed by a dimeric interaction *via* a loop-loop interaction (129, 131). Such different outcomes given the same capping residue elucidate the molecular basis of substrate selectivity that can be achieved in different ways in priming KSs.

The remaining residues that form the acyl-binding pocket are mostly hydrophobic, corresponding well with the mostly hydrophobic priming units observed in PKS. However, priming unit specificity is often more promiscuous *in vitro* than observed *in vivo*. *In vitro*, ZhuH can prime with acetyl-, butyryl-, or isobutyryl-CoA; DpsC shows almost no preference between the same three acyl-CoAs; ecFabH also accepts propionyl-CoA; mtbFabH accepts palmitoyl-, myristoyl-, lauroyl-, decanoyl-, and octanoyl-CoA (73, 76, 125, 130). These results are in contrast with what is observed *in vivo*, where there is a more distinct preference, with the R1128 decaketide showing 85% priming with propionyl, and the decaketide formed by the daunorubicin system is always primed with a propionyl group (11, 104, 114). The observed specificity in FAS can be a physiological phenomenon. For example, ecFabH recognizing propionyl-CoA *in vitro* is not really relevant *in vivo*, because the *E. coli* strains used in most *in vivo* studies naturally lack the ability to biosynthesize propionyl-CoA. In the above case, the difference between *in vitro* and *in vivo* results is a physiological one, not a difference in how the enzymes behave *in vitro versus in vivo*. However, for type II PKSs, because nearly all of *in vivo* studies were conducted in *S. coelicolor*, which is capable of providing propionyl-CoA, we can consider an alternative explanation, and one explanation for this promiscuity is pocket plasticity as observed in homologous structures, inhibitor binding assays, and molecular dynamics simulations. Past ecFabH structures show significant disorder in regions making up the binding pocket when a ligand is not present and molecular dynamics simulations support this (132, 133). Additional support for priming KS plasticity comes from experimental work where covalent inhibitors still bound to the mtbFabH active site event after mutation to block the entrance (134). Finally, biochemical, structural, and molecular dynamics experiments with the priming KS PqsD from *Pseudomonas aeruginosa*, which condenses and cyclizes anthranilic acid and decanoic acid, indicate a conformational change to large substrate entry, similar to mtbFabH (135, 136).

One way that the promiscuity is likely overcome *in vivo* is through an additional partner to the priming KS. The next most common enzyme in an initiation module after the priming KS is an additional AT protein that enhances starter unit fidelity. Concurrent with the discovery of DpsC as a priming KS, the putative acyl transferase DpsD was identified as a potential candidate that contributes to propionate priming, although functional studies in heterologous hosts indicate that DpsD is dispensable (11, 117, 118). However, cell free reconstitution studies give mixed conclusions as to whether DpsD is needed for propionate priming. Later, *in vitro* assays reveal that DpsC is promiscuous with short acyl-CoAs, suggesting that DpsD enhances the priming unit fidelity of DpsC, though no work to elucidate DpsD alone has been completed yet (117, 118, 125).

In addition, ZhuC, a DpsD homolog from the R1128 system, has a clear role in priming unit selection, acting as an editing TE that removes short chain acyl groups from ACP, reducing acetate priming (137). Editing TE activity was also confirmed in the enterocin system for the protein EncL, which removed acetate from ACP much quicker than benzoate, the expected priming unit (138). Additional homologs of DpsD, ZhuC, and EncL also exist in the frenolicin and aclacinomycin PKSs (FrnK and AknF), which could also serve as editing TEs. Therefore, there remains significant fruitful research that can be performed to better understand these unique AT-like proteins.

However, not all putative ATs are editing TEs. GilQ from the gilvocarcin system actively loads ACP with propionyl-CoA, and without GilQ present *in vivo*, propionate-primed gilvocarcin is not produced (139). Interestingly, the gilvocarcin system does not contain a priming KS despite of its nonacetate priming, which shows the flexibility present in the initiation module and some resemblance to type I PKSs (140). Additional homologous AT proteins (ChryQ and RavQ) are found in the chrysomycin and ravidomycin systems, which also lack a priming KS, yet they prime with propionate (141). This suggests a class of initiation module centered around an actively loading AT protein, which holds the possibility of enhancing priming unit fidelity in other systems. At the time of writing, further work on ChryQ and RavQ has not been performed; therefore, there is no confirmation that they have the purported loading AT function.

There are additional possible enzyme complexes that are involved in priming unit selection (142, 143). For example, EncN is involved in transferring a free fatty acid immediately to ACP with no CoA-linked intermediate (144, 145). Another enzyme, AuaEII, generates a CoA-linked substrate for the PKS. (143). The AuaEII crystal structure was solved successfully, and is similar to previously reported aryl-ligase structures (146). These enzymes offer yet another dimension to incorporate new priming units into polyketides, and could provide a robust platform for novel priming unit entry into polyketide biosynthesis.

In summary, when a polyketide produced by a type II PKS that is nonacetate primed, an initiation module composed of one or more enzymes is present to effect nonacetate priming. The most common enzyme in this module is the priming KS, which can be divided into FabH-like (Cys-His-Asn), or DpsC-like (Ser-His-Asp) based on their catalytic triad. FabH-like priming KSs catalyze the Claisen condensation between a priming unit and malonyl-ACP, while DpsC-like priming KSs also can directly transfer the priming unit to ACP that in turn transfers the priming unit to the KS/CLF for extension. Priming KSs have a priming unit preference, but do not have a strict specificity, requiring additional factors to achieve synthetic fidelity. Editing TEs such as ZhuC, EncL can aid in enforcing priming preference by removing acetate from ACP, thus reducing the amount of acetate reaching the KS/CLF active site (137, 138). Additional systems such as GilQ, EncN, and AuaEII (with AuaE) can provide precursors to the PKS systems (139, 142, 143). To date, there are very few structures for these enzymes, with structures only available for ZhuH, DpsC, and AuaEII (123, 125, 146). Fortunately, these structures are complemented by many homologous structures in systems outside of PKS research. However, there remains significant work to structurally elucidate nonacetate priming in the systems covered in this section. One example of this is for the putative active loading ATs of which GilQ is the only one with biochemical support (139). Therefore, a structural understanding of substrate preference in these priming enzymes is a much needed next step to advance our understanding of the enzymes responsible for priming unit selection, so that we can build a robust foundation for future engineering.

Despite the relative lack in structural information for enzymes involved in priming unit selection, there have been successful engineering attempts to diversify priming unit incorporation. Early combinatorial biosynthesis used the R1128 initiation module (ZhuH, ZhuC, and cognate ACP ZhuG) with the actinorhodin or tetracenomycin minimal PKS (ACP, KS/CLF, and MAT), with additional enzymatic transformation by the actinorhodin KR (ActKR), actinorhodin ARO/CYCs, or R1128 ARO/CYCs (147). The initiation module successfully primed the polyketide chains with propionyl or isobutyryl to produce 10 new compounds that had varied chain lengths (Act or Tcm KS/CLF) and cyclization (Act or Tcm ARO/CYCs). Further work used the frenolicin minimal PKS in conjunction with the R1128 initiation module, producing two different polyketide chain lengths, and altering priming unit composition when cultures were supplemented with amino acids that could be catabolized (148). This resulted in a further four compounds produced, including one primed with 2-methylbutyryl-CoA, indicating acceptance by ZhuH. The authors further report more than 40 more possible molecules based on predicted products from combinations they had not performed.

A second example of successful engineering was with the enterocin system *in vivo* and *in* vitro. In the *in vivo* project, the authors inactivated the *encP* gene responsible for the first step in L-phenylalanine conversion to a benzoyl moiety, leaving EncN as the only path for priming unit entry (149). Subsequently, cultures were fed with various aryl acids, the most successful of which where *p-*fluorobenzoate, cyclohex-1-enecarboxylate, and 2- and 3-thiophenecarboxylate. This work was expanded upon in a later *in vitro* project where the same aryl acids were added to a reconstitution of individually purified enzymes with the other necessary synthetic components (150). Aryl acid addition combined with the presence or absence of EncM, catalyzing oxidative carbon skeleton rearrangement, 10 new wailupemycin F or G derivatives and a new p-chlorobenzoate-primed product (not including its epimer) were produced, indicating expanded synthetic flexibility *in vitro*, hypothesized by the authors to be due to an editing enzyme not included in the reaction mixture. In total, twelve unnatural priming units were well-incorporated by EncN across both studies, supporting further engineering opportunities if greater system modifications are made. These examples show how powerful engineering the primer unit selection can be, and individual protein engineering has not yet been employed, leaving more opportunity for novel compound production through a protein engineering approach.

### Biosynthetic logic: chain elongation in type II PKSs

**Figure 4:**
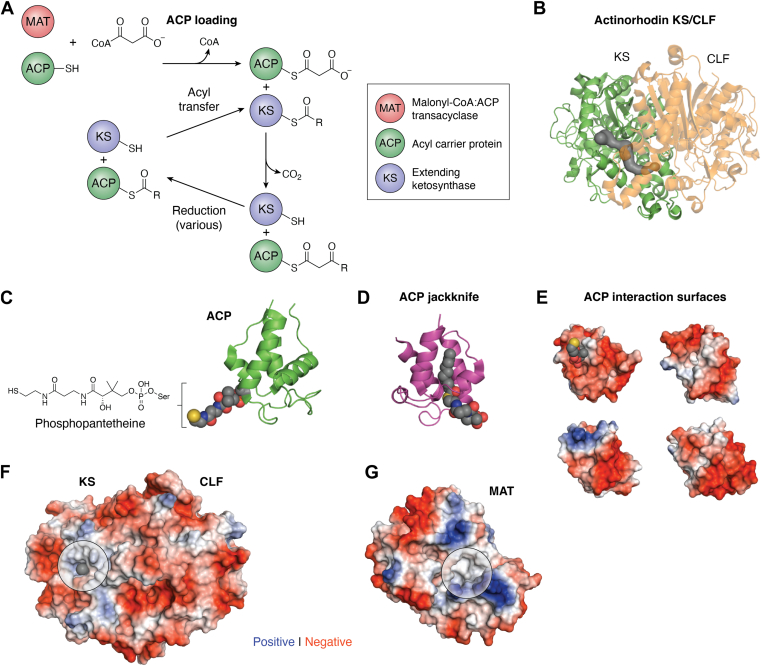
**The molecular logic of elongating enzymes in type II PKS.***A*, chemical reaction catalyzed by the type II polyketide ketosynthase/chain length factor (KS/CLF, PDB code 1TQY). *B*, the crystal structure of actinorhodin KS/CLF shows a long substrate binding pocket that extends from the active site to the KS/CLF heterodimer interface, enabling KS/CLF to elongate the polyketide until it reaches 16 carbons (octaketide). *C*, the NMR structure of acyl carrier protein (ACP, PDB code 2AF8) with the active site serine covalently links to the phosphopantetheine group. *D*. the interior pocket of *Escherichia coli* fatty acid ACP bound with the octanoyl group, showing how the interior pocket can protect the growing intermediate, supporting the “jackknife” mechanism. *E*, the negatively charged protein surface of ACP, which can form extensive electrostatic interactions with (*F* and *G*) the positively charged protein surface of ACP partners such as the KS/CLF and MAT (the active site entrances are shown in *circle*). PKS, polyketide synthase; MAT, malonyl-CoA:ACP transacylase; KS/CLF, ketosynthase/chain length factor; PDB, Protein Data Bank

The foundation of type II polyketide biosynthesis lies at elongation, where the polyketide is lengthened by multiple condensations with one (or additional) extender units to build the polyketide chain. In most type II PKSs, chain extension is mainly performed iteratively by the minimal PKS, which consists of ACP, KS/CLF, and MAT (Fig. 4*A*) (151). The end product is a highly reactive poly-β-ketone that will spontaneously cyclize without enzymatic guidance, as observed in *in vivo* and *in vitro* reconstitutions (16, 17, 84, 85, 152, 153). The KS/CLF is an obligate heterodimer formed between a catalytically active KS and an “inactive” KS, which lacks catalytic residues (Fig. 4*B*) (72, 154, 155, 156). In rare cases, chimeric KS/CLF formed from one part from two systems are functional, but CLF monomers are generally not compatible with KS monomers from different PKS systems, indicating differences at the dimeric interface that preclude chimeric KS/CLF as an engineering path (157, 158). Unlike the priming KS that has a Cys-His-Asn catalytic triad, the extending KS contains a Cys-His-His catalytic triad with an additional conserved lysine residue that is required for efficient polyketide chain extension (102). Despite the difference in catalytic residues, the condensation reaction mechanism is the same as in priming KSs, where a malonyl moiety is decarboxylated to form a nucleophilic enolate, resulting in a Claisen condensation with the KS cysteine-bound polyketide intermediate (Fig. 4*A*) (66). Repeated condensations extend the chain into an amphipathic channel that is formed by the KS/CLF dimeric interface and is primarily responsible for determining the number of extensions (Fig. 4*A*) (156, 159, 160). In order to change the polyketide chain length, mutations of residues that defined this amphipathic channel resulted in polyketides with altered length, while the ability to incorporate longer priming unit traded off the number of extensions until the resulting polyketide chain reached the certain chain length (as defined by the original KS/CLF) (82, 161). Currently, there are only three KS/CLF structures reported, due to difficulty in expressing and purifying the KS/CLF from the host organism where it is most stably expressed (Fig. 4*B*) (156, 159, 160). The three structures are largely in agreement with the KS fold, active site residues, and dimeric interface area. The main difference lies in the CLF size, with actinorhodin CLF consisting of 383 residues while the aryl polyene CLF from *Xenorhabdus doucetiae* is only 211 residues.

The protein partner of KS/CLF is ACP, which is a relatively small protein compared to the catalytic enzymes in type II PKS, at approximately 90 residues in size (Fig. 4, *C* and *D*). It forms a four helix bundle that contains a conserved serine residue, which is phosphopantetheinylated from CoA, the prosthetic group that forms a thioester linkage with the polyketide intermediate or the typical extender unit malonate (Fig. 4*A*) (162, 163, 164). The thioester linkage allows for a reversible transthioesterification that enables intermediate transfer from ACP to the KS active site cysteine that is required for performing another extension step. The PPT group also enables ACP to protect the intermediate during the synthetic process. This protection is achieved in FAS biosynthesis *via* a “jackknife” mechanism through which the hydrophobic intermediate folds into the hydrophobic core of ACP, shielding the thioester bond from hydrolysis (Fig. 4*D*) (162, 163, 164). Though this is not possible in polyketide biosynthesis due to the polar nature of the intermediates, these intermediates (linear or cyclic) may lay flat on the ACP surface, thus reducing exposed surface area between transfers to the next biosynthetic enzyme (162, 163, 164).

Critical to its role in interacting with different enzymes during polyketide synthesis, ACP has a negatively charged surface, while enzyme domains that need to interact with the ACP in PKS and FAS have complementary positively charged patches at their surface (Fig. 4, *E* and *F*) (56, 160, 165, 166, 167, 168, 169, 170, 171, 172). This strongly suggests that ACP interacts with its PKS partners through charge-charge interactions. The ability to swap ACP between systems further supports charge complementarity as the primary interactive mechanism. However, some combinations of ACP and type II PKS enzyme domains are incompatible, resulting in no product formation (173, 174, 175, 176). This incompatibility shows that ACP-partner interactions are more complicated than simple charge-charge interactions, and that protein surface complementation between ACP and its partners should also be necessary. It has been further identified that ACP acts dynamically with its partner proteins, with interactions primarily centered around helix II, where the PPT prosthetic group is attached at the N terminus (Fig. 4*C*) (166, 169). Therefore, while ACP swapping remains a viable option to create chimeric PKS systems, there is now sufficient information to fine tune ACP-partner interactions for improved synthetic throughput and fidelity.

The final member of minimal PKS is MAT, which is responsible for loading malonyl onto ACP, though this is not absolutely required because some PKS ACPs can self-malonylate *in vitro* (177, 178, 179). In type II PKS gene clusters, an MAT is not commonly present, yet polyketide production is consistently observed, even in heterologous organisms, strongly indicating that the MAT that is responsible for loading ACP with malonate is from the FAS (101, 160, 180). The discovery of crosstalk between FAS and PKS led to the structure of *S. coelicolor* MAT, the second MAT structure to be solved, and careful investigation biochemically *via* mutation and kinetics (177, 181). The structure was similar to the *E. coli* MAT solved earlier and gave insight into why methylmalonyl-CoA was not accepted as a substrate; M126 and F200, located at the end of the substrate-binding pocket, sterically block ɑ-substituents. The subsequent biochemical experiments support a malonyl-CoA preference over methylmalonyl-CoA, but not by M126 and F120. Wild type and some mutants (A197D, V98Q) self-acylated with methylmalonyl-CoA at a catalytic efficiency five orders of magnitude less than malonyl-CoA, and could also transfer to ACP (177). However, hundreds of structure-based *S. coelicolor* mutants were generated by the Stanford team, but all lost activity without altering the MAT specificity (personal experience of Tsai while she was a postdoc in the Khosla group, unpublished). These results pointed to delicately balanced active site where structural contributions by surrounding residues (not necessarily in the active site) were key for successful catalysis. Since then, the focus has shifted away from engineering the shared MAT to investigate MAT inhibition that can treat infections such as in *M. tuberculosis* (182, 183, 184, 185). Given the strict requirement of MAT for FAS function and therefore organism survival, combined with the significant failure of MAT engineering *in vitro* as observed by the Stanford group, for type II PKSs, the MAT domain is not well-suited for extender unit alteration as the AT domains in type I PKSs.

Engineering attempts for the KS/CLF to alter polyketide chain length and ACP to understand protein-protein interactions (PPIs) have been sparse (82). In the case of KS/CLF, it has proven difficult to achieve successful mix and match dimer engineering because there appears to be general incompatibility between KS and CLF from different systems (82). Mutagenesis-based engineering to alter chain length specificity in the actinorhodin, tetracenomycin, and fredericamycin systems has been moderately successful in comparison. When key CLF residues in the amphipathic channel where the polyketide intermediate binds were mutated, decaketide production was observed for the typically octaketide-producing actinorhodin KS/CLF, and octaketide production for the typically decaketide-producing tetracenomycin KS/CLF (82). However, the unnatural length polyketides were accompanied by significant production of the native polyketide length, comprising approximately one-third of total product in the actinorhodin system and two-thirds of total product in the tetracenomycin system. Similar mutational work was performed using the fredericamycin system, but without any success in producing polyketide lengths that are not defined by the minimal PKS. Forty-eight different mutants were created based on the previous work in the actinorhodin and tetracenomycin systems, but these mutants did not synthesize new polyketides that are different from the wild type system in chain lengths (186). Given the limited success for mutational work, it appears that our understanding of the factors controlling chain length fidelity is limited. It may be that one such important factor includes a competition between Claisen-condensation and ACP-binding, which requires a more detailed model of the dynamics between ACP and KS/CLF.

The main engineering attempt for ACP has been a systematic swapping of regions between the actinorhodin ACP and the *E. coli* ACP (167). A total of sixteen different swaps were made, eight swaps of *E. coli* ACP regions into Act ACP, and eight *vice versa*. Each swap included one or more of the core four helices and connecting loop regions, fully covering all sections of ACP. Most notably, replacing the actinorhodin ACP helix II with helix II from *E. coli* ACP enabled binding to *E. coli* FabF that was not possible before, though with seven to 10 times worse binding than the native interaction. This points to a more complex binding dynamic that includes interactions across different ACP helices, along with the enzymatic partner dynamics alluded to earlier, which will require more subtle changes to achieve ACP compatibility with nonnative enzymes (166).

### Biosynthetic logic: Chain reduction in type II PKSs

**Figure 5:**
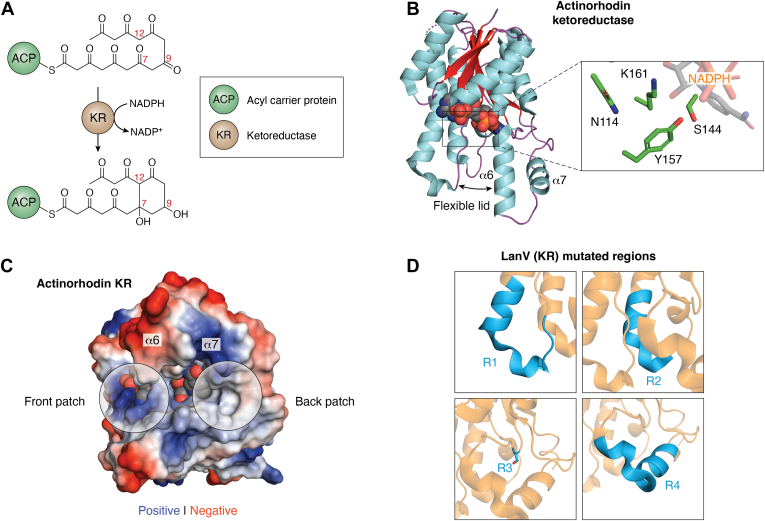
**The molecular logic of ketoreductase (KR) in type II PKS.***A*, the chemical reaction catalyzed by the type II KR, using the actinorhodin KR (ActKR) as an example that conducts first cyclization at C7-C12 and ketoreduction at C9. *B*, side view of the actinorhodin ketoreductase (ActKR). α-helices are colored *cyan*, β-strands *red*, and loops *purple*. NADPH is represented as spheres with carbons colored *gray*. The lid region is formed by α-helices 6 and 7 which can take an open or closed conformation. The open conformation is depicted in this image. The motif TGxxxGxG is located near the cofactor NADPH. *C*, the two entrances to the KR active site are circled in *green*. The front and back patches of ActKR are *circled*. *D*, LanV, a tailoring ketoreductase involved in landomycin biosynthesis. The largest difference in structure is at the lid region where LanV has a truncated lid in comparison to ActKR. PKS, polyketide synthase; KR, ketoreductase.

After the full poly-β-ketone chain is produced *via* the extending enzymes in type II PKS systems, an early acting NADPH-dependent KR, if present, most PKSs commonly reduce the ninth carbonyl group to an alcohol, such as in the actinorhodin PKS (12, 176, 187, 188, 189, 190). KRs have a well-studied Rossman fold, consisting of a twisted parallel β-sheet sandwiched by α-helices, where NADPH binds to a conserved TGxxxxG motif at the C terminal ends of the β-sheet (Fig. 5, *A* and *B*) (189, 191, 192, 193). The core catalytic residues form a Tyr-Lys-Ser-Asn tetrad that facilitates proton transfer to bulk solvent after a stereospecific hydride attack on the ketone from the nicotinamide ring (Fig. 5*B*). This proposed proton transfer mechanism is supported by the presence of crystallographic waters in multiple KR structures (192, 194). The active site cleft is comprised of residues from the α-helices flanking the central β-sheet and includes two α-helices (ɑ6-ɑ7) that form a lid-like structure with a flexible hinge (Fig. 5*B*). This results in an open, flexible active site architecture with no indication of how the linear substrate could be constrained to enforce highly specific reduction (commonly at C9), nor whether early acting KRs can distinguish between intermediates with different chain lengths. For example, the ActKR can reduce poly-β-ketones ranging from hexaketides (12 carbons) to dodecaketides (24 carbons), and the hedamycin KR (HedKR or HedE) reduces tetraketides, undecaketides, and dodecaketides while the daunorubicin C9 KR, DpsE, cannot reduce octaketides but can reduce decaketides (18, 21, 22, 95, 176, 195, 196). In addition to ketone reduction, the C9-reducing KR such as the ActKR is also tightly associated with C7-C12 first ring cyclization, though it remains unclear how the KR facilitates ring formation despite high quality biochemical and structural studies (83, 197, 198, 199, 200).

Current biochemical and structural work for C9-reducing KRs has used ActKR as the primary model for understanding the three aspects described above: stereospecific reduction, substrate specificity, and cyclization. Early work uncovered complementary evidence regarding the order of reduction and first ring cyclization. ActKR was found to favor bicyclic over linear compounds, with trans-1-decalone and α-tetralone showing activity *in vitro*, supporting that linear poly-β-ketone cyclization occurs before C9 reduction, congruent with earlier molecular docking (192, 201). In the molecular docking results, C7-C12 cyclization positioned C9 closest to the nicotinamide ring for reduction, providing a reasonable mechanism for consistent C9 reduction (192). Next, ActKR and HedKR stereospecificity was probed using an oxidation assay with enantiomerically pure α-tetralol that allowed for assessing KR stereoselectivity. ActKR preferred S-(+)-tetralol over R-(−)-tetralol at roughly 3:1 while HedKR was only active for S-(+)-tetralol, pointing to a consistent S-configured preference across distantly related KRs (189, 202). Intensive mutational work based on comparison to the stereochemical motifs of type I polyketide KR, combined with the ɑ-tetralol stereochemical assays and *in vitro* reconstitution, resulted in developing a sophisticated model for poly-β-ketone binding, positioning, C7-C12 cyclization and C9 reduction in the active site (202, 203). In this model, the negatively charged ACP first interacts with positively charged residues (R38, R65, and R93) high on one side of the active site cleft (Fig. 5*C*). The linear intermediate then enters underneath the gating residues located at the top of the cleft (P94 and M194) and is positioned close to NADPH with hydrophobic steering residues (V151, F189, and V198) within the cleft. Cyclization is enabled by hydrogen bonding between the T145 sidechain and the C11 substrate carbonyl to depress C12 α-proton ${pK}_{a}$, catalyzing intramolecular aldol condensation to produce the C7-C12 ring. As a result of cyclization, the C9 carbonyl is then positioned closest to NADPH, and hydride transfer produces an S-configured alcohol.

However, it remained unclear how an S-configured alcohol is consistently produced. In actinorhodin synthesis, C9, after ketoreduction to an alcohol, is aromatized *via* dehydration of the C7-C12 ring, thus preventing stereochemical assignment at C9. The prevention of alcohol loss by removing the ARO/CYCs produces the C9 alcohol-containing mutactin, but stereochemical assignment has been unsuccessful despite using multiple methods (202, 204). This issue is further compounded by an unclear understanding of how ACP binds to the KRs, which could affect the side (front or back, Fig. 5*C*) of the planar C9 carbonyl that gets reduced, producing different stereochemistry at the alcohol. The two models of ACP binding are (1) binding to the positively charged patch (the “front” side; R38, R65, R93) comprised of three arginine residues as mentioned earlier, or (2) binding to the opposite side (the “back” side) of the active site cleft either on top of the lid helices (ɑ6-ɑ7) at the tetrameric interface or near another group of charged and polar residues (Q149, R220, and N260) (Fig. 5*C*) (192, 198, 205).

The most recent work to investigate the ACP-KR interaction used a combination of protein-protein docking and NMR to model ACP binding to the front side, where the three arginine residues are located (206). After creating the ACP-KR models, the researchers performed extensive molecular dynamics work, using a C7-C12 cyclized polyketide intermediate attached to their KR-ACP model, and found that S-favored reaction poses were massively dominant over R-favored poses. This is in line with prior observations that mutating the key arginines reduced the binding of ACP to ActKR, supporting the ACP-KR interaction at the “front” side. Supporting this hypothesis, the stereochemical assays shows that ActKR prefers S-(+)-tetralol (203). Therefore, front patch binding is more reasonable than the back side binding, although direct experimental evidence remains lacking due to the transient nature of KR-ACP interactions, the lack of easy cross-linking between the active sites of KR and ACP, and the reactive nature of the natural poly-β-ketone substrate.

Besides the C9-reducing KRs that act on ACP-bound substrates, there exists a second class of KRs that act on polyketide intermediates after ring cyclizations and release from ACP (207). Such KRs stereospecifically reduce at C15, C17, C19, or C21. Sequence alignment shows that these KRs are well-differentiated between each type, as well as with the C9 KRs (208, 209, 210, 211). They share the same Rossman fold as the C9-reducing KRs, but they have a significantly shortened lid region (Fig. 5*D*) (212, 213, 214, 215). Due to the less reactive nature of their native substrates, cocrystal structures with substrate analogs have been successful and show similar positioning to the emodin-bound ActKR structure (201, 212, 215). Such substrate analogs that bind in the KR active site provide ample information with which to engineer the change in stereospecificity.

Engineering in the tailoring KRs was attempted using LanV and CabV, reducing hydroxylated prejadomycin to 11-deoxylandomycinone (11-DLM) and reducing a doubly hydroxylated prejadomycin to gaudimycin C, respectively (208, 216, 217). Four major regions of differences between the two KRs were identified: R1 consists of residues 102 to 118 that make up part of the dimeric interface and bottom of a loop opposite the lid; R2 covers residues 154 to 164 that are spatial neighbors of R1, part of the dimeric interface, and a few residues near the active site cleft. R3 is a single residue (S192) that is analogous to F189 in ActKR, and R4 contains residues 198 to 210 that constitute the lid region. Swapping the R3 region between LanV and CabV had the largest impact in changing the resulting product, but swapping all four regions was required to create the largest product change (213). In the wild type LanV, 11-DLM was produced, but with the chimeric LanV, it biosynthesized a 4:1 ratio of gaudimycin C to 11-DLM. Chimeric CabV had a much larger change with 11:1 production of gaudimycin C to 11-DLM converted to a 1:16 production. Although the region swap did result in a stereochemical flip for both enzymes (11-DLM and gaudimycin C have opposite C15 stereochemistry), no unnatural stereochemistry for the native substrate were observed, because substrate specificity changed with stereospecificity. Given the identical product orientation in the cocrystal structures, the authors hypothesized that the aromatization of angular ring occurs in 11-DLM production but not in gaudimycin C production, which in turn rotates the C15 carbonyl plane, thus positioning the rings to favor the opposite stereochemical outcome.

Similar mutational work in the daunorubicin KR (DpsE) and ActKR have also been successful in changing the product and stereochemical outcome. In ActKR, a comparison was made with type I KRs, which have well-defined sequence motifs that determine the stereochemical outcome. The motif in ActKR was identified as PGG (residues 94–96), a motif similar to the B-type KR LDD motif (202, 218). Subsequent single, double, and triple mutants identified the P94L mutation as capable of swapping the 3:1 ratio of S-(+)-tetralol to R-(−)-tetralol selectivity to completely S-(+)-tetralol selective with identical catalytic efficiency to the wild type ActKR (202). Another single mutant, V151L, was only active for R-(−)-tetralol, with 2.6 times the catalytic efficiency of wild type for the same substrate and 75% the catalytic activity of wild type for S-(+)-tetralol (203). Unfortunately, it could not be confirmed whether these mutations were effective in swapping stereochemistry within the native poly-β-ketone substrate during *in vitro* reconstitution, because chiral HPLC and chiral shift reagent NMR results were ambiguous (202).

Studies of DpsE sought to uncover why it could not reduce the octaketides produced from the actinorhodin minimal PKS, but it could reduce the decaketides from the tetracenomycin minimal PKS as mentioned earlier (22). Two chimeric DpsE proteins were created; one by swapping the lid region (residues 217–246; ActKR numbering) of DpsE with the lid region of ActKR and the other by swapping the lid and mutating G94, A152, and L153 in DpsE to P, V, and H to match the residues in ActKR (219). Both chimeric DpsE proteins gained the ability to produce the octaketide mutactin when added to the actinorhodin minimal PKS, matching the expected outcome if the native ActKR was used. The chimera with two swaps was more effective, but the lid swap made the largest difference in mutactin production. The above result helps identify regions that are important for the observed substrate specificity.

In summary, significant KR engineering has been achieved based on detailed investigative work. In both C9-reducing KRs and late-reducing KRs, flips in stereochemical outcome have been achieved through careful mutations. However, successful stereochemical changes have not yet been observed for C9-reducing KRs in the context of PKS reconstitution, nor have late-reducing KRs had substrate selectivity and stereochemical outcome decoupled. These stand as the next barriers to a bright future in engineering these KRs for novel, accessible production of polyketides with alternative stereochemistry.

### Biosynthetic logic: The aromatization and cyclization of type II PKSs

**Figure 6:**
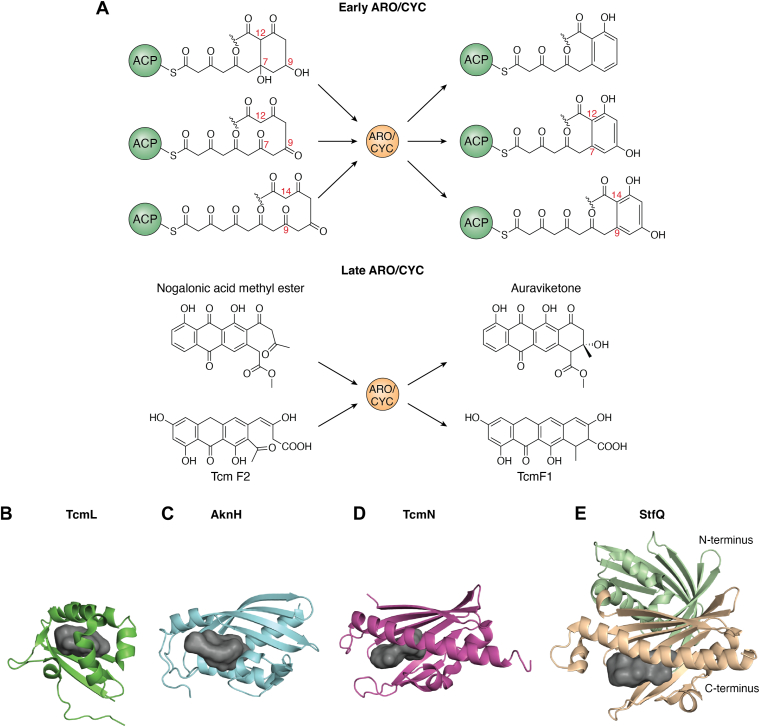
**The molecular logic of aromatase/cyclase (ARO/CYC) in type II PKS**. *A*, chemical reactions catalyzed by early ARO/CYCs and late-stage ARO/CYCs that create aromatic rings. *B*-*E*, visual comparison of the ARO/CYC folds solved to date. TcmI (*B*) and AknH (*C*) are fourth ring cyclases with folds that differ from the early acting ARO/CYCs. TcmI has a very shallow pocket defined on the sides by 3 α-helices and a β-strand while the back of the pocket is formed from a β-sheet. AknH, and its related fourth ring cyclase SnoaL, have a larger pocket into which the entire intermediate can fit. The antiparallel β-sheet forms one side with two short α-helices bordering the pocket entrance with two longer α-helices forming the rest of the pocket. The monodomain TcmN (*D*) and didomain StfQ (*E*) take helix grip folds where an anti-parallel β-sheet forms the back of the pocket, and a long α-helix closes it off. Loops at the β-sheet ends form the entrance to the pocket. In all proteins, key polar and basic residues position the intermediate in the active site and catalyze site specific ring closure. Hydrophobic residues define the pocket shape to fold the intermediate to favor a particular cyclization pattern. PKS, polyketide synthase.

The final step in the biosynthesis of the core polyketide molecule is aromatization and cyclization catalyzed by aromatase and cyclase (ARO/CYC), which creates the general molecular shape. Currently, RO/CYCs that catalyze the aromatization and cyclization early in the synthetic process can be categorized under five main categories as follows (Fig. 6*A*).

1. No KR is present; monodomain catalyzes C7-C12 cyclization (ZhuI (93))
2. No KR is present; monodomain catalyzes C9-C14 cyclization (TcmN, WhiE ARO/CYC (15, 152))
3. No KR is present; didomain catalyzes C7-C12 cyclization (StfQ, MtmQ (220, 221))
4. KR is present and catalyzes C7-C12 first ring cyclization and C9 reduction. Didomain catalyzes aromatization (BexL, actVII (20, 222))
5. No KR is present; multiple ARO/CYCs collaboratively produce a discoid molecule (Rem cyclases (223))

In the Tsai lab, structures for ARO/CYCs acting early in the biosynthesis for categories 1 to 4 have been solved. The structures of TcmN, ZhuI, WhiE ARO/CYC, StfQ, and BexL all show a helix-grip fold for the ARO/CYC domain that consists of a 7-stranded antiparallel β-sheet that is closed off by a single long α-helix (Fig. 6, *C* and *D*) (15, 93, 152). The residues inside this pocket where a polyketide intermediate can bind are composed of a mixture of hydrophobic and polar residues, and the pocket size is correlating with intermediate chain length. WhiE ARO/CYC (24 carbons) has the largest pocket, and ZhuI (18–20 carbons) has the smallest pocket (Fig. 6, *C*–*E*). A conserved arginine residue is located at the back of the pocket for all active domains (N terminus of didomain BexL, C terminus of didomain StfQ) and mutational analysis shows that it is critical for either substrate positioning or activity. Mutating R69 to alanine in TcmN abolished C9-C14 cyclization activity, instead producing C7-C12 cyclized compounds, while the R66A mutation in ZhuI abolished cyclization activity completely (15, 93, 152). Equivalent mutations in WhiE ARO/CYC, StfQ, and BexL led to a significant reduction in activity rather than complete (220, 221). A conserved acidic residue is also present in the BexL N terminus and ZhuI ARO/CYC that was identified as also important for cyclization activity (15, 93, 152). Further mutational work guided by molecular docking using precyclized molecules provides a strong support for acid-base mechanisms that drives cyclization and aromatization of the linear intermediates. The ARO/CYC structures provide significant insights into cyclization specificity as directed by the ARO/CYC.

In addition ARO/CYC enzymes can act later during synthesis to further catalyze ring closure, without which cyclization patterns can vary (Fig. 6*A*). Examples of late-acting ARO/CYCs include TcmI (Fig. 6*B*), AknH (Fig. 6*C*), SnoaL, JadI, and PgaF. TcmI, AknH, and SnoaL, all catalyze C2-C19 fourth ring cyclization in tetracenomycin, aclacinomycin, and nogalamycin biosynthesis, respectively (224, 225, 226). In addition, AknH and SnoaL produce opposite chiral centers in their respective products, a chemical feature not typically conserved for other ARO/CYC products due to ring aromatizations that convert all carbons to trigonal planar electron geometry. Meanwhile, JadI and PgaF are part of an interesting class of fourth ring cyclases, because they are responsible for angling the fourth ring in angucycline compounds. However, JadI is strictly dependent on JadE, a KR, and JadD, a possible first ring aromatase, to produce the typical angled fourth ring, otherwise cyclization is spontaneous and the fused ring carbon skeleton is not produced (227). PgaF is similar in catalyzing second, third, and fourth ring closures after prior hypothetical C9 reduction, C7-C12 cyclization, and first ring aromatization performed by a KR and ARO/CYC, though it is uncertain whether these additional enzymes are required as is true for JadI (228). Given the difference in the resulting carbon skeleton shape, linear and angled fused ring systems, these ARO/CYCs present an appealing target for engineering.

The crystal structures of the late acting cyclases TcmI, SnoaL, and AknH are solved and show two different protein folds. TcmI represents one of the folds and has a ferredoxin-like shape, with a four strand anti-parallel β-sheet at its dimeric interface and three α-helices forming the outside layer (Fig. 6*B*) (229). A sulfate ion was found in the pocket near H26, R40, and H51, while the rest of the pocket was comprised of hydrophobic residues. Mutational analysis revealed that H26, D27, R40, and H51 were keys for fourth ring closure, and their positioning led the authors to hypothesize that the active site residues mediate substrate binding rather than catalyzing the reaction, and that water could act as the acid-base catalyst. The structures of SnoaL and AknH share structural elements with TcmI, but they have a five rather than four strand antiparallel β-sheet forming a tetrameric interface (Fig. 6*C*) (230, 231). Two long α-helices are located on one face of the β-sheet to form a pocket between the two secondary structure elements, while two short α-helices and a loop between strands four and five flank the opening to this pocket, making it deeper. The polycyclic products, derived from the closely related nogalonic acid methyl ester and aklavinone, were successfully cocrystallized in the active site for both proteins and showed fourth ring closure or opening, respectively. Such results, supported by strong electron density, point to the shared catalytic mechanism between these two ARO/CYCs (230, 231). The pocket residue composition is similar to TcmI, containing mostly hydrophobic residues with key polar or charged residues located near ring A, where ring closure took place. Structure-based mutagenesis identified invariant D121 in both proteins that is required for catalytic activity, and two residues located near ring A, Y15/F15 and N51/L51, are found to play a role in C9 stereochemical outcome. The Y15F and Y15F/N51L in AknH mutants, matching SnoaL in those positions, produced 20% and 50% S-configured C9 alcohols, respectively, compared to all R-configured. The structural and biochemical data support an acid-base catalyzed aldol condensation to mediate ring closure. The authors conclude that ARO/CYCs can influence stereochemical outcomes in polyketide biosynthesis (224, 225).

Engineering involving ARO/CYCs has focused on swapping ARO/CYCs into heterologous systems to control cyclization and aromatization outcomes for polyketides with different chain lengths (15, 17, 19, 21, 95, 147, 197). In such engineering attempts, the inherent capabilities of the ARO/CYC are essential to achieve the desired outcome. However, this does not always work, because of poor ACP-ARO/CYC interactions during domain swapping, leaving the difficult challenge of increasing ACP-ARO/CYC compatibility across systems (22, 232, 233). Mutational work to interrogate the catalytic mechanism of early acting ARO/CYC, as well as the AknH double mutant that matches the corresponding residues in SnoaL, are two closest studies aiming to engineer individual ARO/CYCs. Despite extensive mutations, the only significant changes achieved have been to abolish the C9-C14 cyclization of TcmN (Fig. 6*D*), leading to C7-C12 cyclization instead and a partial switch in stereochemistry at C9 from R to S while achieving only 50% wild type activity; likewise, the mutations of StfQ did not result in new cyclization patterns (Fig. 6*E*) (92, 231). Therefore, much more remains to be learned about the cyclization specificity and catalysis of ARO/CYC, and compatibility with heterologous PKS components to achieve greater engineering success.

## Conclusions and future

PKS research has advanced substantially since the actinorhodin gene cluster was first cloned into a heterologous host that successfully produced actinorhodin (9). Subsequent work in the late 80s and 90s consisted of dissecting different PKS gene clusters to elucidate gene function followed by attempts to produce novel polyketides by mixing and matching from multiple PKS systems. The early 2000s were then marked by a distinct transition to *in vitro* reconstitution over *in vivo* reconstitution due to the power of recombinant DNA technology. Complementary to this shift, the completed *S. coelicolor* A3(2) genome sequence was published in 2002 and revealed that significantly more BGCs were present in microbes than was previously thought (234). This discovery opened up new avenues in genome mining to produce and characterize new secondary metabolites from less active, or inactive BGCs. Thanks to the mining efforts, more enzymes of type II PKS domains were sequenced and cloned, which in turn provided more targets for crystallization. Consequently, the crystal structures for the type II MAT, priming KS, extending KS/CLF heterodimer, C9-reducing KR, first ring cyclase, and fourth ring cyclases were solved, providing high-quality structural information about every core synthetic step of type II PKSs (92, 123, 156, 181, 192, 198, 229, 230, 231). Meanwhile, the crystal structures for mammalian and bacterial type I FASs were reported at around the same time, revealing, for first time, structural insights into these megasynthases (61, 62). Although the megasynthase structures are from type I enzymes, they offer some insights about PPIs that are still applicable to the type II PKSs.

The drastic reduction in sequencing costs led to a huge increase in bacterial genome and metagenomic sequencing availability, in turn motivating computational tool development for the prediction of BGCs, and databases to store annotated gene clusters became readily available (33, 35, 37, 38). With such powerful tools and databases now at our disposal, it is now easier than ever to efficiently find BGCs that contain unique enzymes for further study. Incredible resources are now available to conduct PKS research effectively at all levels: finding and manipulating genes, expressing individual proteins, as well as structural and biochemical characterization.

However, these advances have yet to surmount old challenges, and much remains to be uncovered before engineering can consistently result in the product yield and fidelity similar to the native systems. The two critical barriers currently facing type II PKS research are the highly reactive nature of the linear intermediates and the transience of PPIs during type II polyketide biosynthesis. The core biosynthetic logic for type II PKS is iterative extension of a poly-β-ketone. Such an intermediate is prone to spontaneous cyclization, which has been observed through varied products biosynthesized by minimal PKS systems (16, 80, 81, 83, 84, 84, 85). This precludes the possibility of structural characterization using the native linear substrate for the KS/CLF, KR, and ARO/CYC. Without structural information about substrate positioning, developing a mechanistic understanding and defining substrate specificity requires rigorous mutation as demonstrated by studies of ActKR (202, 203). Such studies lack further insights needed to fully understand the catalytic mechanism and substrate specificity, which stymies engineering attempts at the protein level. The answer to this problem has been to generate substrate analogs, primarily S-N-acetylcysteamine thioester mimics over the years (235). More chemically analogous mimics have also been used, with the most recent employing isoxazole or oxetane moieties (236, 237). Yet each of these mimics has only been successfully employed twice so far, leaving the majority of their potential for interrogating PKS active sites untapped (237, 238).

Given the nature of PKS as a multidomain enzyme complex, PPIs are key to achieve an end product with high fidelity. Unlike type I systems where all domains are covalently linked, type II PKSs have separate monodomain or didomain proteins that interact to synthesize a polyketide (63). This makes detailed structural investigations of PPIs incredibly difficult, because weak complexes do not crystallize well, and larger complexes make NMR interpretation difficult. So far, the primary focus of PPI research has been on the interactions of ACP with the synthetic enzymes such as KS domains, since it is the hub of both type I and II PKS biosynthesis. Based on extensive research, it is clear that the basis for ACP interaction with other PKS enzymes is between the negatively charged region on ACP around the conserved phosphopantetheinylated serine and positively charged patch near the partner enzyme active sites (56, 165, 166, 167, 170, 172, 181, 192). Currently, the most effective way to capture the ACP interaction interface structurally has been to develop a mechanism-based cross-linking moiety, load it on to the PPT arm of ACP, then incubate the modified ACP with the desired partner enzyme. This has worked to great success with the type II FAS in *E. coli*, producing ACP-linked structures for the extending KSs FabF and FabB, along with the dehydratase FabA (168, 171, 239). These structures have elucidated that ACP has extensive protein-protein interface with its partner enzymes.

The three main successes in determining ACP interactions structurally for PKS systems include ACP cross-linked to the type I iterative PKS CTB1, the incredibly fortunate cocrystal structure of the KS/CLF-ACP with a bound linear intermediate from *Photorhabdus luminescens*, and the ACP-MCAT cocrystal structure from the same system (160, 240). These structures have given further detailed insight into the ACP-enzyme interface, but the key trend in charge complementarity identified from earlier FAS studies remains. Engineering work building from these structures has yet to emerge, so it is unclear what their impact toward successful engineering in type II PKS systems is at this time.

To this date, there are only two related studies that have attempted to build a multienzyme type II pKS complex model containing more than just ACP and another PKS enzyme, while using this model to investigate the molecular basis of product specificity. Both used a yeast two hybrid system to assess pair PPI and then built a partial model using current structural knowledge for the daunorubicin PKS (241, 242). The best models developed with the data collected were a complex of DpsAB (KS/CLF) with a homology modeled DpsD (putative AT of unknown function) and a complex of DpsD, DpsE (C9 KR), and DpsY (cyclase) (242). This shows how limited our knowledge is about how PPIs may affect a multienzyme type II PKS complex, especially if we try to link PPI to product fidelity.

There are other studies that also highlighted the importance of PPIs to product fidelity. In the enterocin PKS, the requirement of the C9-reducing KR EncD to be present, an intriguing requirement pointing to a structural contribution to synthetic competence not seen in other systems (243). Similarly, the importance of PPIs in the PKS complex can be observed through the presence of both KR and ARO/CYC that affect the polyketide chain length. This has been observed in the addition of ARO/CYC TcmN to the frenolicin minimal PKS, resulting in only nonaketide production rather than the natural octaketide and nonaketide mixture (17). More examples include the requirement of WhiE ARO/CYC for achieving full-chain extension, and the addition of griseusin ARO/CYC to the minimal tetracenomycin PKS producing a nonaketide instead of a decaketide (95, 244). Chain length alterations involving a KR was observed when disruption of the *otcD1* gene containing an ARO/CYC and ketoreductase produced shorter polyketides of multiple lengths (245). The above studies support the importance PPIs that mediate the observed product specificity.

In conclusion, since the actinorhodin gene cluster was cloned in 1984, a robust understanding of the core catalytic activities required for type II polyketide has been developed with a strong pipeline for BGC discovery in a genomic era. However, production of “unnatural” NPs with the expected benefits to human health have fallen short of early expectations as barriers to type II PKS engineering have been discovered. Great progress has been made to overcome these, yet the two that remain for type II PKS, intermediate reactivity and understanding PPI, will be the future focus to tackle. A better understanding of the above two areas is paramount for reaching the next level in type II PKS engineering where multiple lines of engineering can be combined to achieve higher production levels and fidelity. To this end, an aggressive application of the already existing substrate mimics will greatly enhance our understanding of catalytic mechanism and specificity, while a special effort will be required to continue investigating PPI so as to combine protein and cluster engineering effectively.

## Conflict of interest

The authors declare that they have no conflicts of interest with the contents of this article.
